# A Rare Coexistence of Arteria Lusoria, Truncus Bicaroticus, and Klippel-Feil Syndrome: A Five-Year Follow-Up and Procedural Considerations

**DOI:** 10.7759/cureus.115640

**Published:** 2026-09-02

**Authors:** Silvia Puglia, Evy Micieli

**Affiliations:** 1 General Internal Medicine, Private Practice, Zürich, CHE; 2 Angiology, University Hospital of Zürich, Zürich, CHE

**Keywords:** aberrant right subclavian artery, arteria lusoria, congenital aortic arch anomaly, dysphagia lusoria, klippel-feil syndrome, kommerell diverticulum, non-recurrent laryngeal nerve, subclavian artery supply disruption sequence, transradial catheterisation, truncus bicaroticus

## Abstract

Arteria lusoria, an aberrant right subclavian artery (ARSA) arising as the most distal branch of the aortic arch, is the most common congenital aortic arch anomaly, yet it is clinically silent in over 90% of individuals and is usually discovered incidentally. We report the incidental identification of ARSA together with a truncus bicaroticus and Klippel-Feil syndrome (KFS) in a 32-year-old man investigated for intermittent pretracheal pressure. Contrast-enhanced MRI of the neck demonstrated a retro-oesophageal ARSA with mild oesophageal compression and revealed congenital fusion of the C2-C3 and C6-C7 vertebrae, consistent with KFS, together with suspected canal stenosis at C4-C5. Thoracic MR angiography confirmed ARSA with proximal ectasia at its origin, absence of a Kommerell’s diverticulum (KD), and a truncus bicaroticus, alongside congenital right-sided latissimus dorsi and serratus anterior atrophy. Serial MR angiography over five years demonstrated no significant interval progression, and a conservative surveillance strategy was adopted in line with current guidelines. The coexistence of ARSA, truncus bicaroticus, and KFS in this patient raises the possibility of a shared developmental basis. Previously described embryological mechanisms, including the subclavian artery supply disruption sequence (SASDS), provide a plausible framework linking abnormalities of pharyngeal arch development and cervical somite segmentation during weeks 4-8 of gestation; however, such a relationship cannot be established from a single case. Beyond surveillance, the principal clinical message is procedural: unrecognised ARSA carries potentially avoidable risks during right transradial catheterisation, thyroid and anterior cervical surgery (through its association with a non-recurrent laryngeal nerve), and oesophageal, mediastinal, and thoracic procedures. Recognition of one component of this constellation may therefore warrant consideration of associated congenital anomalies and targeted vascular imaging, particularly when invasive cardiac, cervical, or thoracic procedures are planned.

## Introduction

Arteria lusoria, derived from the Latin *lusus naturae* (“trick of nature”), refers to an aberrant right subclavian artery (ARSA) arising as the most distal branch of the aortic arch rather than from the brachiocephalic trunk. This vascular anomaly results from abnormal involution of the right fourth pharyngeal arch artery and the proximal right dorsal aorta during weeks 4-8 of embryogenesis. Consequently, the artery crosses the midline to supply the right upper limb, most commonly following a retro-oesophageal course (approximately 80% of cases) [1]. Despite its relatively frequent occurrence, ARSA is clinically silent in over 90% of individuals and is typically identified incidentally during imaging or invasive procedures. When symptomatic, it may cause dysphagia or dyspnoea due to compression of adjacent structures [2,3]. The prevalence of ARSA ranges from 0.4% to 2.2% in the general population, with a female predominance; these estimates derive largely from autopsy and cross-sectional imaging series [2,3]. Increased prevalence has been described in individuals with chromosomal abnormalities such as trisomy 21 and Turner syndrome [4,5]. The reported co-prevalence with truncus bicaroticus (an anatomical variation in which the left and right common carotid arteries share a single common origin rather than separate origins from the brachiocephalic trunk and aortic arch, respectively) of 19-29% [3] is drawn from selected ARSA series and imaging cohorts and probably exceeds the frequency in unselected populations. Emerging data also suggest an increased prevalence of thoracic aortic aneurysm in patients with ARSA [6], and in a cohort of patients with heritable and non-heritable arteriopathies, ARSA was associated with additional congenital anomalies [7]. A clinically relevant variant is Kommerell’s diverticulum (KD), an enlargement at the origin of the aberrant artery. KD is associated with risks of aneurysmal degeneration, dissection, rupture, and thromboembolic events [6].

Klippel-Feil syndrome (KFS), characterised by congenital fusion of cervical vertebrae, has historically been estimated to occur in approximately 1 in 40,000-42,000 live births, although imaging-based studies in selected populations suggest that clinically silent cervical fusion may be considerably more common; KFS is frequently associated with congenital cardiovascular anomalies [8]. However, the coexistence of ARSA and KFS remains poorly characterised in the literature. We report a case of incidental identification of ARSA, truncus bicaroticus, and KFS in a single patient and discuss the hypothesis of a potential shared embryological mechanism and the clinical and procedural implications of their coexistence.

## Case presentation

A 32-year-old man with no regular medications and a remote history of ventricular septal defect presented in January 2020 with intermittent pretracheal pressure. Biopsy of a hypoechoic pretracheal nodule was consistent with a reactive lymph node. Extensive haematological and infectious work-up, comprising immunofixation, serum free light chains, immunoglobulins, full blood count, and metabolic panel, together with viral serology (HIV, hepatitis B, and hepatitis C) and nucleic acid testing (Epstein-Barr virus (EBV), Cytomegalovirus (CMV), and Herpes simplex virus (HSV)), was unremarkable aside from mildly elevated ferritin and borderline lymphocytopenia, with no monoclonal band on immunofixation (Table 1).

**Table 1:** Selected laboratory findings relevant to the diagnostic evaluation. Results are shown with their units. Hyphen (-) denotes not applicable. Ag/Ab: Antigen/antibody; anti-HCV: Hepatitis C antibody; CMV: Cytomegalovirus; EBV: Epstein-Barr virus; HBsAg: Hepatitis B surface antigen; HSV: Herpes simplex virus.

Laboratory test	Result	Reference range
Lymphocyte count	0.99 × 10⁹/L	1.1-3.5 × 10⁹/L
Ferritin	418 µg/L	30-400 µg/L
CRP	<0.6 mg/L	<5 mg/L
Serum immunofixation	No monoclonal band	-
HIV Ag/Ab	Negative	Negative
HBsAg	Negative	Negative
Anti-HCV	Negative	Negative
CMV PCR	Not detected	Not detected
EBV PCR	Not detected	Not detected
HSV-1/2 PCR	Not detected	Not detected

**Figure 1:**
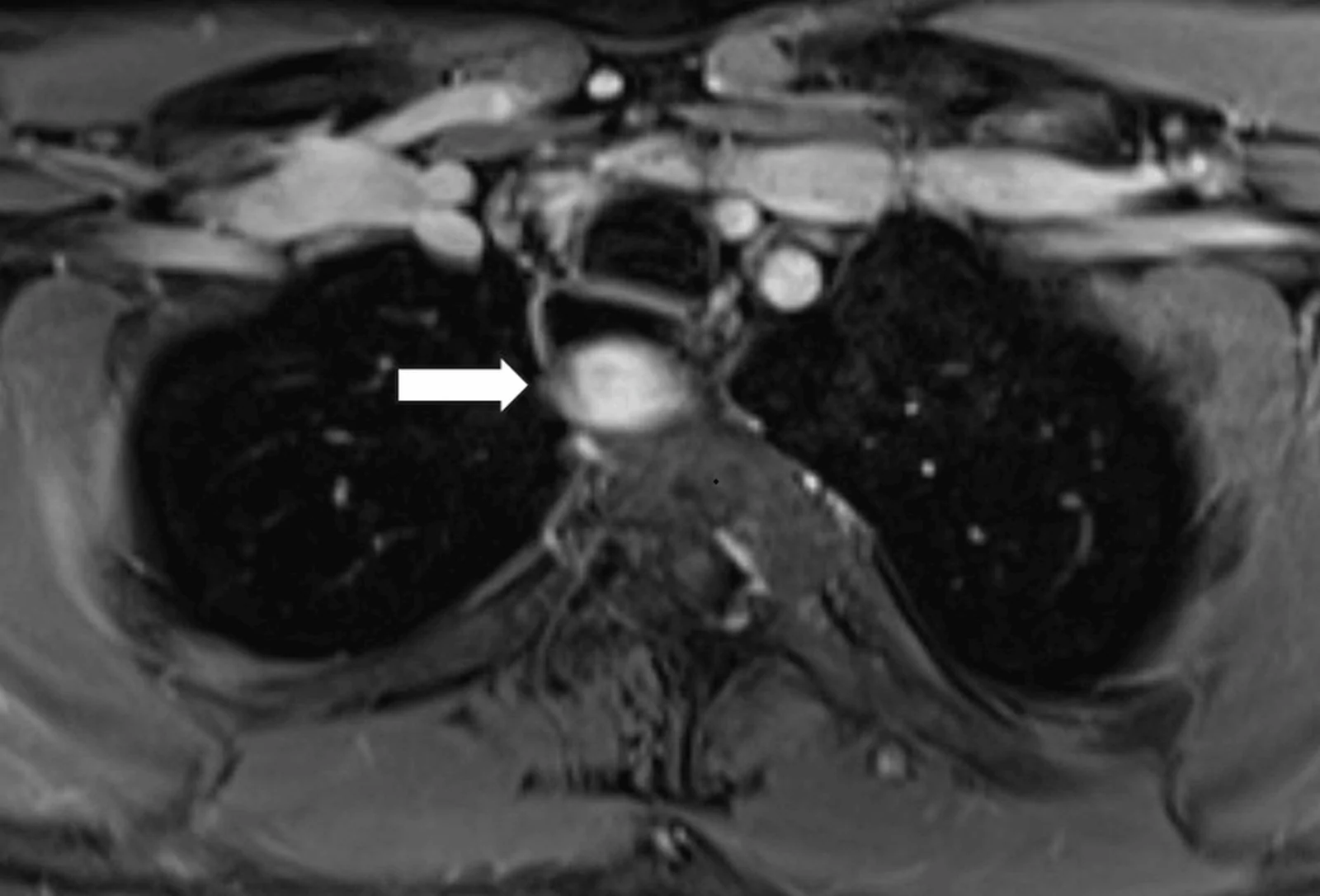
Axial T1-weighted MRI at the thoracic inlet showing the retro-oesophageal course of the aberrant right subclavian artery between the oesophagus and the vertebral column, with its potential compressive effect (white arrow).

The patient was discharged with a diagnosis of reactive lymphadenopathy. One year later, owing to persistent symptoms, the patient underwent further evaluation. Laboratory tests revealed only borderline lymphocytopenia. Contrast-enhanced MRI of the neck identified an aberrant right subclavian artery with a retro-oesophageal course and mild oesophageal compression, while excluding lymphadenopathy (Figure 1).

The same imaging study demonstrated Klippel-Feil syndrome with block vertebrae at C2-C3 and C6-C7, as well as suspected cervical canal stenosis at C4-C5. Additional incidental findings included post-traumatic rib changes and a partial empty sella. ENT reassessment was unremarkable. Angiological evaluation revealed a systolic blood pressure asymmetry between the arms (132/89 mmHg right vs 152/90 mmHg left), consistent with the distal origin of the aberrant vessel (Table 2).

**Table 2:** Vital signs and bedside measurements.

Parameter	Result	Reference range	Unit
Heart rate (at transthoracic echocardiography)	73	60-100	bpm
Blood pressure, left arm	152/90	-	mmHg
Blood pressure, right arm	132/89	-	mmHg
Radial pulses	Palpable bilaterally, right mildly reduced	-	-

**Figure 2:**
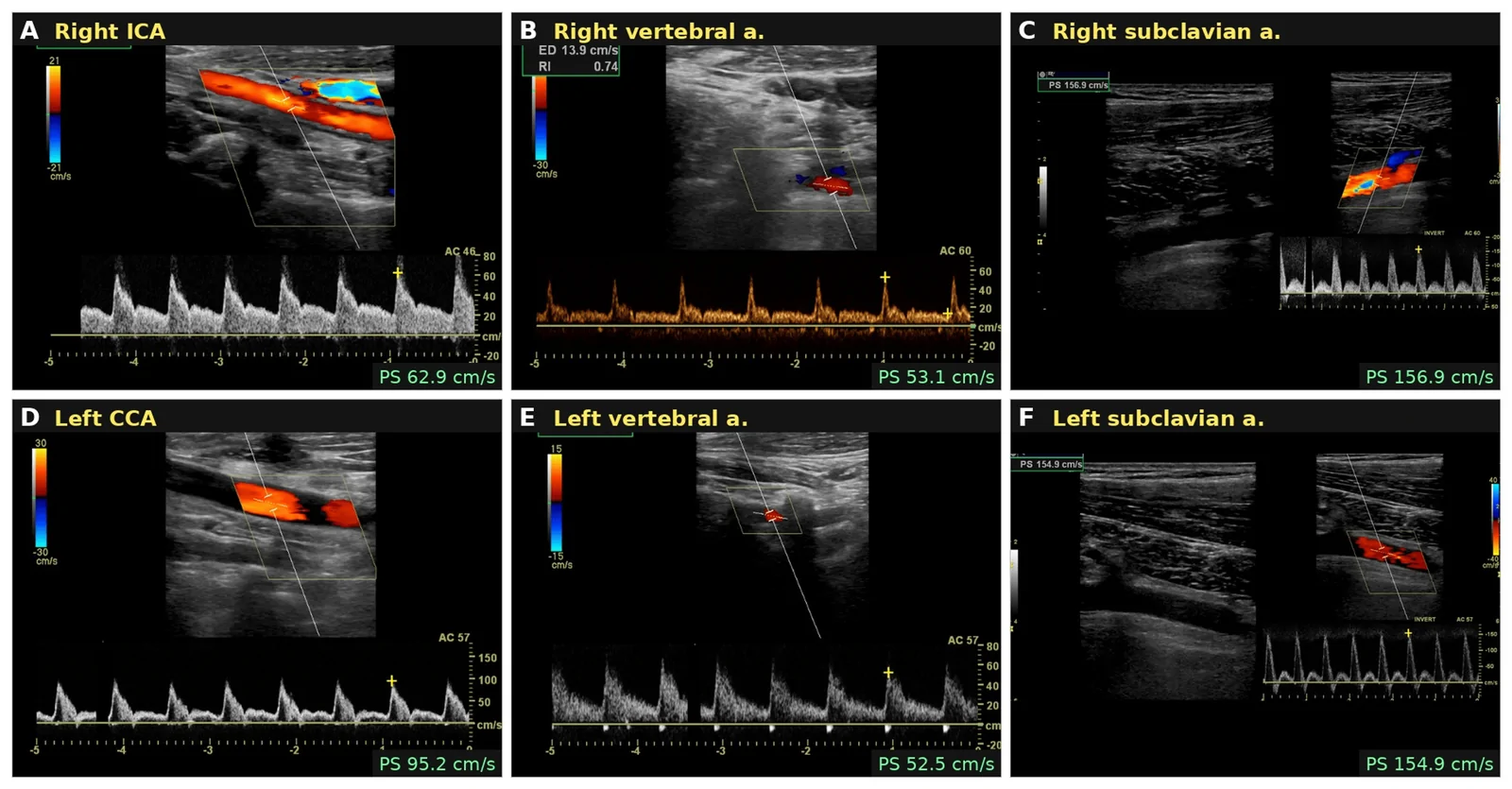
Colour-coded duplex sonography of the supra-aortic arteries demonstrating patency of all interrogated vessels without haemodynamically significant stenosis. (A) Right internal carotid, (B) right vertebral, (C) right subclavian, (D) left common carotid, (E) left vertebral, and (F) left subclavian artery, each shown with colour Doppler and the corresponding spectral waveform; peak systolic velocity is annotated in each panel. The carotid arteries show expected spectral profiles, the subclavian arteries show normal triphasic profiles, and both vertebral arteries are patent with antegrade flow. The proximal segment of the aberrant right subclavian artery (arteria lusoria) lies in the posterior mediastinum and is not accessible to cervical sonography.

Duplex ultrasound confirmed patency of the supra-aortic arteries without significant stenosis (Figure 2).

**Figure 3:**
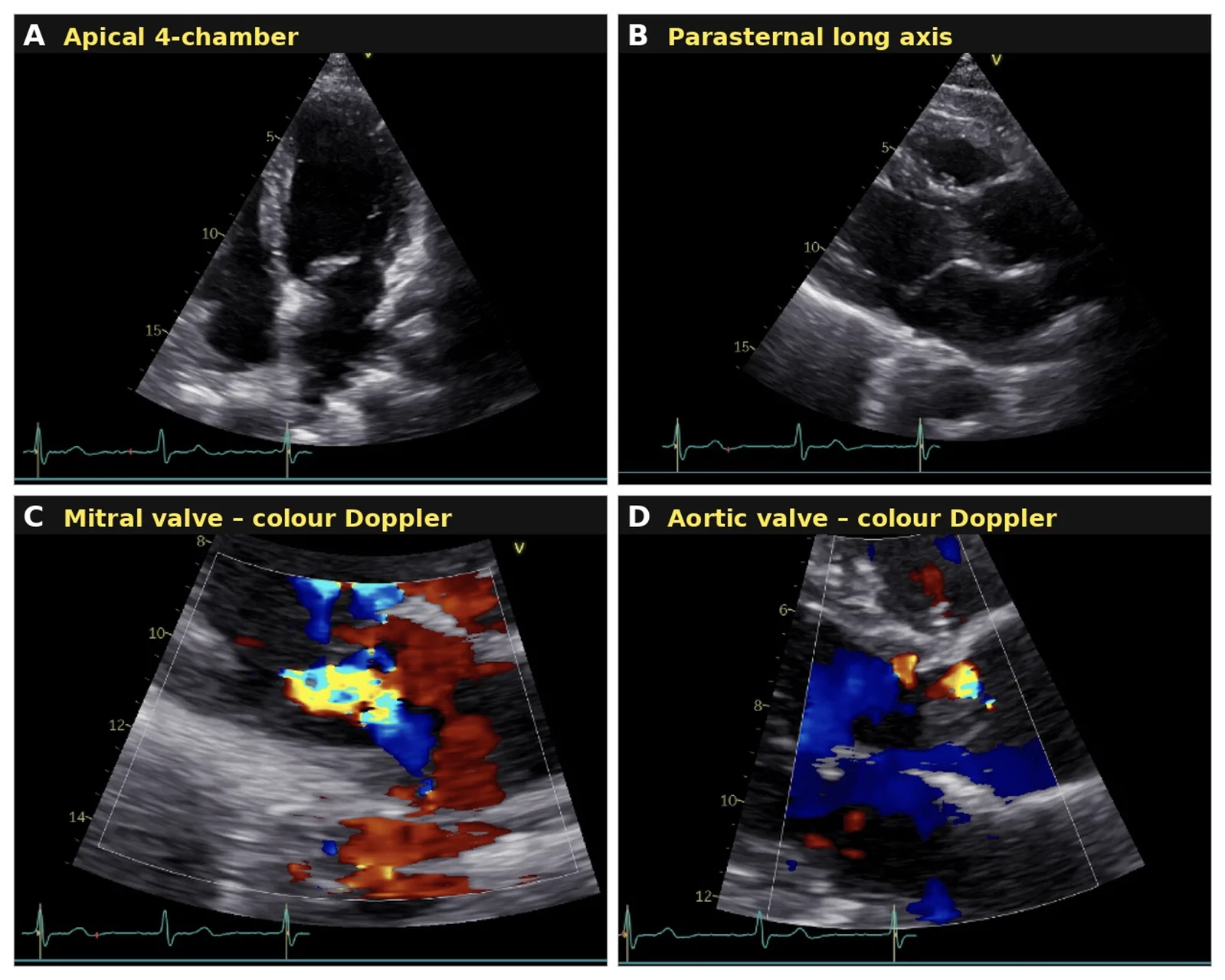
Transthoracic echocardiography. (A) Apical four-chamber and (B) parasternal long-axis views demonstrating a non-dilated, non-hypertrophic left ventricle with preserved systolic function (biplane ejection fraction, 58%) and a mildly dilated left atrium. (C) Colour Doppler at the mitral valve showing a regurgitant signal consistent with minimal mitral regurgitation in the setting of meso- to telesystolic prolapse of both leaflets, and (D) colour Doppler at the aortic valve showing mild commissural insufficiency of a tricuspid aortic valve.

Transthoracic echocardiography showed preserved left ventricular function, minor valvular abnormalities, and mild left atrial enlargement (Figure 3).

**Figure 4:**
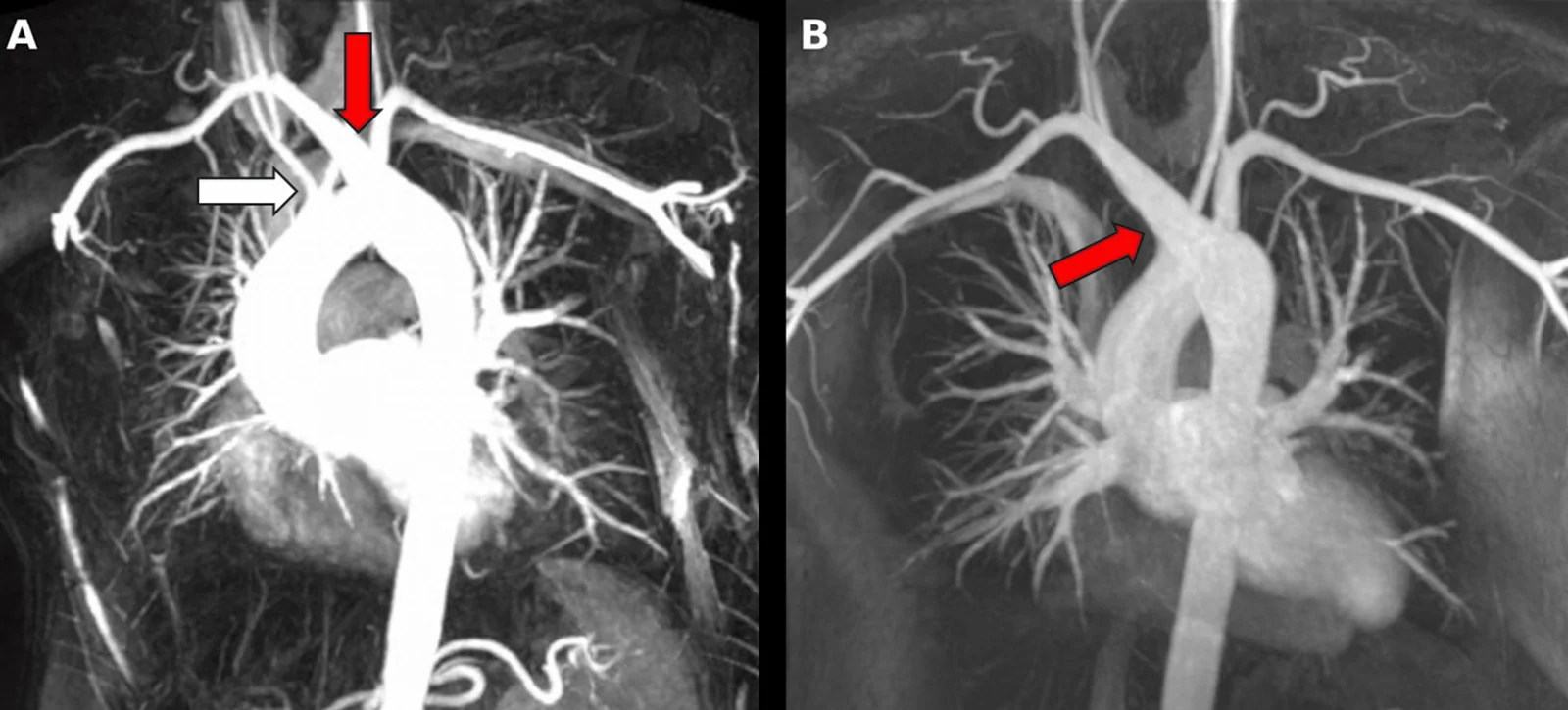
Maximum intensity projection (MIP) MR angiographic images, anteroposterior and rotated oblique views. (A) Aberrant right subclavian artery (red arrow) and associated truncus bicaroticus (white arrow). (B) Proximal ectasia of the arteria lusoria and its relationship to the descending aorta (red arrow).

**Figure 5:**
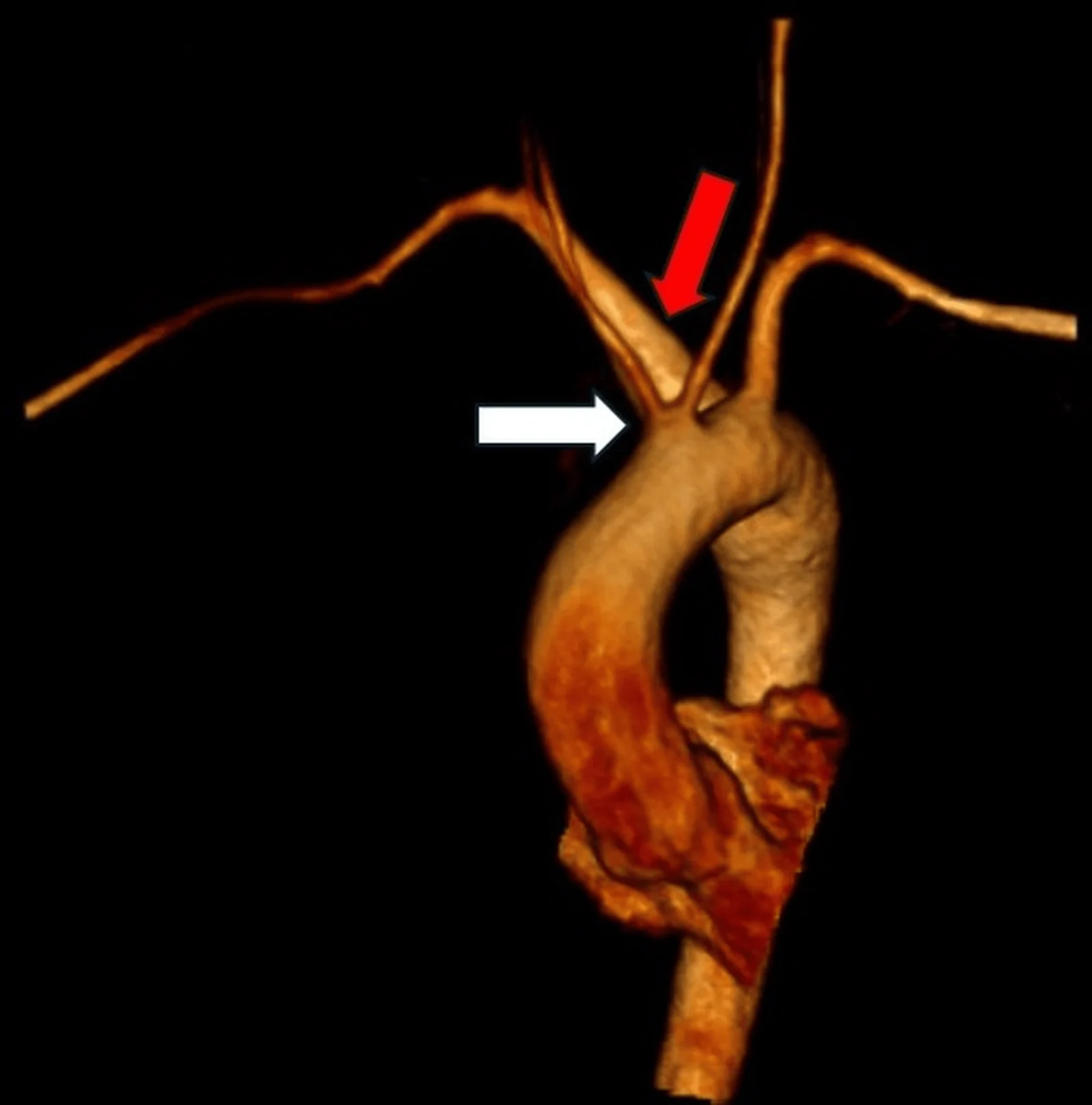
3D volume-rendered reconstruction of the thoracic aorta and supra-aortic vessels, right lateral oblique view. Showing the aberrant right subclavian artery arising as the last branch of the aortic arch and looping posteriorly across the midline in its retro-oesophageal trajectory (red arrow), and the truncus bicaroticus as a shared origin of both common carotid arteries (white arrow).

Thoracic MR angiography further characterised the vascular anatomy, demonstrating ARSA with proximal ectasia measuring 22 mm (defined as dilatation <150% of the expected normal diameter, as no ARSA-specific diagnostic threshold exists in the literature); absence of a Kommerell’s diverticulum and the presence of a truncus bicaroticus. Notably, right-sided atrophy of the latissimus dorsi and serratus anterior muscles was observed, consistent with a congenital developmental pattern. Aortic dimensions were otherwise normal (Figures 4-5).

Management and follow-up

Given the absence of significant symptoms and the lack of aneurysmal dilation, a conservative approach with serial MR angiographic surveillance was adopted. Follow-up imaging in 2022 confirmed no significant interval progression of the vascular findings, allowing extension of the surveillance interval. In 2024, MR angiography showed ARSA-origin ectasia measuring 25 mm, within measurement variability and below surgical thresholds; no Kommerell’s diverticulum was present, and thoracic aortic dimensions remained stable across serial imaging, within the limits of measurement variability. Repeat imaging in 2026 again confirmed radiological stability.

Serial thoracic aortic and ARSA-origin dimensions across the surveillance period are summarised in Table 3.

**Table 3:** Serial thoracic aortic and aberrant subclavian artery dimensions on surveillance MR angiography. All values are maximum diameters in millimetres measured on thoracic MR angiography. Dimensions showed no true interval progression across the surveillance period, remaining within the limits of measurement variability; no thoracic aortic aneurysm was present at any time point, and the aberrant subclavian artery origin remained below the 30 mm diameter threshold at which repair may be considered. Hyphen (-) denotes a segment not separately measured on that study.

Aortic segment (maximum diameter, mm)	February 2021	February 2022	February 2024	February 2026
Aberrant right subclavian artery origin	22	22	25	24
Ascending aorta	35	35	35	35
Aortic arch, proximal to the left subclavian artery	24	24	24	26
Aortic arch, distal to the left subclavian artery	22	22	22	-
Descending aorta, distal to the aberrant subclavian origin	33	33	33	32
Descending aorta at the level of the left atrium	23	23	23	23

This approach is consistent with current guidelines, which support conservative surveillance in asymptomatic patients below treatment thresholds. Surgical or endovascular intervention should be considered for symptomatic ARSA and/or Kommerell’s diverticulum, irrespective of size (European Association for Cardio-Thoracic Surgery (EACTS)/Society of Thoracic Surgeons (STS) 2024; European Society for Vascular Surgery (ESVS) 2026). In asymptomatic patients, treatment may be considered when the aberrant subclavian artery diameter is ≥30 mm (EACTS/STS 2024; ESVS 2026), when the Kommerell diverticulum orifice is ≥30 mm (American College of Cardiology/American Heart Association (ACC/AHA) 2022; EACTS/STS 2024), or when the combined diameter of the Kommerell diverticulum and adjacent descending aorta reaches ≥50 mm (ACC/AHA 2022; EACTS/STS 2024) or ≥55 mm (ESVS 2026) [9-11]. These differences reflect variation in measurement definitions and intervention thresholds among contemporary guidelines. For an incidentally detected, non-aneurysmal, asymptomatic aberrant subclavian artery, the ESVS 2026 guidelines state that serial imaging surveillance at five-year intervals may be considered beginning at age 40, with closer surveillance considered when dilatation or a Kommerell diverticulum is present [11]. Given the presence of ARSA-origin ectasia in our patient, a closer two-year interval was chosen, and surveillance MR angiography of the supra-aortic arteries is scheduled for 2028. Dedicated cervical spine MRI is indicated if neurological symptoms develop, and oesophageal manometry should be considered if dysphagia progresses.

## Discussion

This case illustrates the incidental detection of ARSA in association with truncus bicaroticus and KFS. The coexistence of these anomalies raises the possibility of a shared embryological origin. The subclavian artery supply disruption sequence (SASDS), proposed by Bavinck JN and Weaver DD, offers one theoretical framework through which this coexistence may be interpreted. Partial or complete interruption of subclavian or carotid flow during weeks 4-8 of gestation generates cascading disruptions in aortic arch patterning, ipsilateral somite segmentation, cervicothoracic skeletal development, and neuromuscular perfusion in parallel [12]. ARSA results from variant regression of the right fourth pharyngeal arch artery; truncus bicaroticus from concurrent absence of the brachiocephalic trunk; and KFS from failure of adjacent cervical somite segmentation [1]. Their occurrence within an overlapping developmental period may provide biological plausibility for a shared origin. A previously reported molecular mechanism relevant to these developmental pathways is variation in GDF6, associated with the vertebral segmentation defects of KFS [13]. No genetic testing was performed in the present patient. In our patient, the stable right-sided latissimus dorsi and serratus anterior atrophy is compatible with a congenital developmental abnormality. Its mechanism cannot be determined from the available clinical and imaging data, but SASDS-related hypoperfusion or denervation represents one possible explanation [12]. The hypothesis of a common embryological basis could have clinical implications, as identification of one anomaly may warrant evaluation for the others. However, a causal relationship cannot be established from a single case.

From a clinical perspective, the importance of ARSA lies primarily in its procedural implications. In right transradial catheterisation, ARSA creates technically challenging anatomy for right radial access. The aberrant vessel forms an acute angle at its aortic origin, and guidewire advancement from the descending to the ascending aorta may fail; reported failure rates reach up to 40% [14]. Excessive wire manipulation in this setting carries a risk of arterial dissection. Serra R et al. described ARSA dissection with mediastinal haematoma as a direct complication of right transradial coronary angiography, a life-threatening event requiring emergency hybrid endovascular repair [15]. Pre-procedural knowledge of ARSA allows planning of alternative access (left radial or femoral) and avoids potentially fatal complications. In this patient, the presence of a truncus bicaroticus adds further complexity, as inadvertent coverage of the trunk during endovascular procedures could compromise cerebral perfusion bilaterally.

ARSA is strongly anatomically associated with a right non-recurrent laryngeal nerve (NRLN). The NRLN exits the vagus at the cervical level and enters the larynx directly, rather than looping under the subclavian artery as the recurrent laryngeal nerve normally does, a configuration associated with the absence of the right subclavian artery from its standard thoracic position [16]. In thyroid and parathyroid surgery, the NRLN carries a pooled injury risk for permanent vocal cord paralysis approximately 3.8-fold higher than that of the standard recurrent laryngeal nerve [17]. Preoperative identification of ARSA on CT or neck ultrasound can reliably predict an NRLN and allows intraoperative neuromonitoring to be planned accordingly [16]. This is directly applicable to our patient, who carries ARSA and is therefore highly likely to have a right NRLN, relevant to any future thyroid, parathyroid, or anterior cervical procedure.

The retro-oesophageal course of ARSA places the vessel in direct proximity to the surgical field of oesophagectomy, mediastinal lymph node dissection, and video-assisted thoracoscopic procedures. Mashru D et al. reported ARSA as a significant intraoperative hazard during robotic oesophagectomy, requiring specific vessel identification and protection strategies [18]. A 2025 case report documented the additional risk of an oesophageal-arterial fistula, a rare but potentially fatal complication, in the setting of oesophageal cancer surgery with ARSA [19]. In all these contexts, preoperative CT identification of ARSA is required for safe surgical planning.

Patients with KFS also warrant particular attention because of the high prevalence of associated cardiovascular anomalies. Comprehensive vascular imaging should therefore be considered prior to any invasive procedure. The 2024 systematic review by Niewchas A et al. of 46 published KFS cases with cardiovascular involvement found that 72% met criteria for congenital heart disease, predominantly ventricular septal defect, coarctation, and aortic arch anomalies, with type III (multilevel) KFS as the most common fusion profile [8]. Paul I et al. recommended subclavian artery imaging before cardiac surgery in all KFS patients [20]. Our case supports this observation: given the high burden of cardiovascular co-anomalies documented in KFS and their tendency to remain clinically silent, aortic arch MR or CT angiography should be considered in KFS patients prior to any invasive cardiac, thoracic, or cervical procedure, where unrecognised arch variants may directly alter surgical planning and safety.

## Conclusions

The coexistence of ARSA, truncus bicaroticus, and KFS in our patient raises the possibility of a common embryological basis, although causality cannot be established from a single case. Long-term imaging stability supports conservative management in the absence of significant symptoms or progressive dilatation. The key clinical message is procedural: recognition of ARSA before cardiac, thoracic, or cervical interventions can prevent potentially serious complications and should inform pre-procedural planning. Identification of one of these congenital anomalies may also warrant consideration of associated vascular or skeletal abnormalities. Further studies are needed to determine whether their coexistence represents a potential phenotypic association and to establish its clinical significance.
